# Silver Lactoferrin as Antimicrobials: Mechanisms of Action and Resistance Assessed by Bacterial Molecular Profiles

**DOI:** 10.1021/acsomega.3c07562

**Published:** 2023-11-22

**Authors:** Maciej Monedeiro-Milanowski, Fernanda Monedeiro, Paweł Pomastowski

**Affiliations:** Centre for Modern Interdisciplinary Technologies, Nicolaus Copernicus University in Toruń, Wileńska 4 Str, Toruń 87-100, Poland

## Abstract

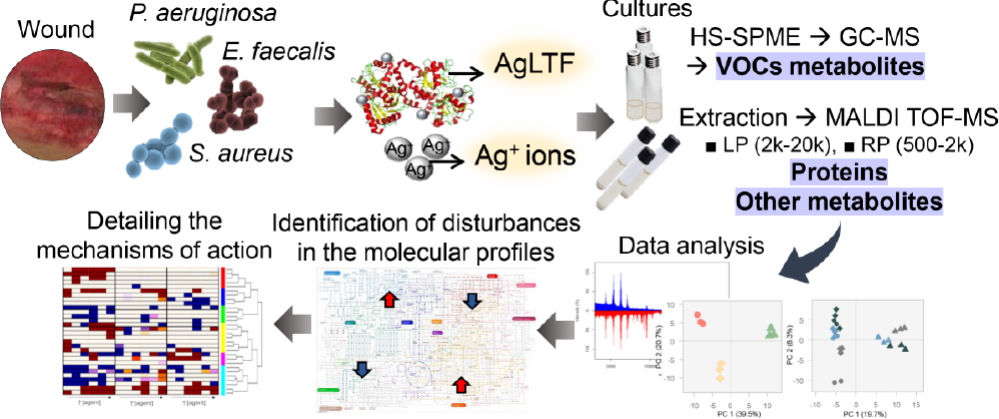

A diverse silver–lactoferrin (AgLTF) complex, comprising silver ions (Ag^+^) and silver nanoparticles, displayed a synergistic antibacterial effect while being almost five times more lethal than LTF alone. Gas chromatography–mass spectrometry and matrix-assisted laser desorption/ionization time-of-flight mass spectrometry—in linear (LP) and reflectron (RP) positive modes—were used to comprehensively analyze metabolites and proteins profiles of bacteria (*Staphylococcus aureus* (SA), *Pseudomonas aeruginosa* (PA) and *Enterococcus faecalis* (EF)) treated using AgLTF complex versus exclusively Ag^+^. Although both agents resulted in similar metabolic shifts in bacteria, AgLTF significantly triggered the production of sulfides (related to bacterial stress resistance), ethanol, 2-butanol (indicating exhaustion of cell respiration), decanoic acid, and nonane (suggesting ongoing oxidative stress). Keto acids formation and fermentation pathways were enhanced by AgLTF and suppressed by Ag^+^. Furthermore, AgLTF appears to interact with proteins fraction of bacteria in a concentration-dependent manner. EF molecular profiles showed less changes between treated and untreated bacteria. On the other hand, SA and PA proteins and metabolic patterns were the most differentiated from untreated bacteria. In conclusion, our study may provide valuable insights regarding the molecular mechanisms involved in AgLTF antimicrobial action.

## 1 Introduction

Microorganisms are able to develop insensitivity against lethal doses of antibiotics known as multidrug resistance (MDR). It causes a major concern for antibiotics regarding the efficacy against pathogen-originated diseases.^1^ The report “Tackling drug-resistant infections globally: final report and recommendations” (2016) states that as many as 10 million people could die annually from antimicrobial resistance by 2050.^2^ Nowadays, MDR represents an alarming societal burden due to additional healthcare expenses.^1^ Although the urgent need for the application of new antimicrobial drugs for clinical use is still present, antibiotic development has slowed dramatically over the past 30 years because of the abandonment of antibiotic discovery programs by many pharmaceutical companies.^3^ Since 2017 only two antibiotics of the eight approved, in fact, were a new chemical scaffold.^4^ Investigations on new antibiotics often require long-term research on the effectiveness and safety of the agents, which are time- and resource-consuming.^5^ Nanotechnology offers opportunities for re-examination of the biological properties of common antimicrobial compounds through manipulation of their size to alter the effect.^6^ Silver nanoparticles (AgNPs) have important biological properties, that is, they are effective bactericidal agents against antibiotic-resistant strains, as well as common fungi including *Aspergillus*, *Candida*, and *Saccharomyces*.^7^ AgNPs are easily synthesizable, the most effective nanoparticles against bacteria and other microorganisms, and are highly biocompatible. To develop resistance against AgNPs, bacteria would have to target multiple parallel mechanisms of action.^5^ New promising agents have emerged that bind silver cations with bioactive ligands to reduce or eliminate their toxic properties. Such effects were reported for AgNPs end-capped by serum albumin and gold nanoparticles formed in lysozyme crystals.^8^ Lactoferrins (LTF) are iron-binding glycoproteins that belong to the transferrin family. They were first isolated from both bovine and human milk in 1960, and since then, they represent a great potential as a natural defense agent. They demonstrate wide antimicrobial activity against a number of bacterial, viral, and fungal pathogens, confirmed by *in vitro* studies.^9^ Native LTFs exert their antimicrobial action through sequestration of iron. However, how the antimicrobial LTF peptides act against the microbes remain undetermined.^9,10^ In the work of Pryshchepa and co-workers, the researchers investigated the sorption process of Ag^+^ onto bovine LTF based on the batch isotherm study. They synthesized a heterogeneous silver–LTF (AgLTF) complex using ammoniacal solution of silver nitrate, known as “Tollens’ reagent.” The reason for the usage of silver complexation with ammonia resulted from the arranged facilitation of silver solubilization in basic conditions to avoid undesirable formation of insoluble silver oxide (Ag_2_O) above pH 6. However, Ag^+^ tends to reduce in the presence of organic compounds with the formation of metallic nanoparticles, and this nanocomposite also comprises partially AgNPs. The AgLTF complex exhibited synergistic antibacterial effect compared with both native bovine LTF and Ag^+^, while being comparable to silver toxicity. The obtained complex appeared to be a promising solution as an antibacterial agent for the treatment of chronic wounds.^11^ The binding of silver cations with bioactive ligands has led to obtain products with eliminated toxic properties of Ag^+^ for interaction with human cells and increased human biocompatibility.^8^

Therefore, the current study proposes an inedited metabolomic approach to obtain a deeper understanding of molecular mechanisms related to the antimicrobial interaction between selected bacteria and AgLTF complex. For such purpose, we carried out experiments employing solid-phase microextraction (SPME) in the headspace (HS) variant combined with gas chromatography–mass spectrometry (GC–MS). This technique allowed to extract, enrich, and detect volatile organic compounds (VOCs) from many biological matrices such as breath, tissues, saliva, and bacteria. They are generated as a consequence of metabolic processes and secreted from cells located in various parts of the body. They include various functional groups including alcohols, aldehydes, hydrocarbons, fatty acids (FAs), esters, and ketones.^12^ Bacterial VOCs are generated as products or byproducts of metabolic pathways. For example, fatty acid biosynthesis causes the emission of hydrocarbons, aliphatic alcohols, and ketones, whereas indole evolves from the breakdown of the amino acid tryptophan. The identification of bacterial volatiles allows to track VOC evolution to be employed as a tool for the diagnosis of disease, with specific VOCs acting as markers for the presence or absence of pathogenic bacteria.^13^ The investigation of bacterial profiles can be related to the presence of a specific strain and its metabolic behavior; hence, the bactericidal effect of various agents was measured by the evaluation of these profiles. Our group confirmed the usefulness of HS-SPME–GC–MS for the identification of microbial species and infections, and to study metabolic changes occurring in bacteria in the face of stressing agents, mostly AgNPs and silver nitrate.^14−16^ Matrix-assisted laser desorption/ionization–time-of-flight mass spectrometry (MALDI–TOF MS) is recognized as a powerful technique for the identification of microorganisms and for the investigation of, for instance, bacterial drug resistance. Protein profiles obtained within the mass range of 2000–20 000 Da using MALDI–TOF MS linear positive mode (LP) can reflect many physiological states of bacteria.^17,18^ On the other hand, reflectron positive mode (RP), within the range of 500–3000 Da, is useful to assess smaller molecules such as metabolites.^18^

The aim of the present work is to use HS-SPME–GC–MS for a comprehensive analysis of the panel of bacterial VOC metabolites allowing to reveal the mechanism of action of AgLTF complex versus Ag^+^ (from silver nitrate) on bacteria isolated from wounds through proposed metabolic manner. Complementary, MALDI–TOF MS method was applied using LP and RP modes of analysis aimed to register both bacterial small proteins and other metabolites. Alterations in identified metabolites were interpreted from the point of view of agent-induced modulations in bacterial pathways, and the study of MALDI–MS spectra led to an additional elucidation of the interaction between the agents and other sets of biomolecules.

## 2 Results and Discussion

### 2.1 Growth Curves and Minimum Inhibitory Concentration

**Figure 1:**
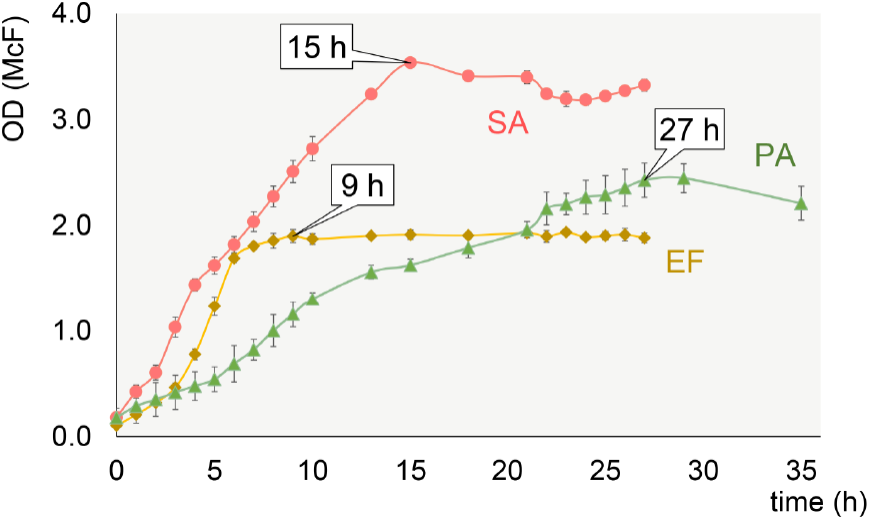
Growth curves obtained for the studied bacteria. The numerals in the boxes indicate the optimal incubation times selected for the further experiments. OD = optical density, SA = *S. aureus*, PA = *P. aeruginosa*, and EF = *E. faecalis*.

Figure 1 presents the growth curves of three selected bacteria grown in Mueller Hinton Broth (MHB) medium. Based on the assessed curves, further experiments were conducted using incubation times of 15, 27, or 9 h for the cultivation of *Staphylococcus aureus* (SA), *Pseudomonas aeruginosa* (PA), and *Enterococcus faecalis* (EF), respectively. The mentioned time points refer to the beginning of the stationary phase (plateau phase). At this stage, the ratio of alive/dead bacteria is rather constant, minimizing the contribution of metabolic alterations due to the growth process. Therefore, when using such cultivation times, the alterations observed in the acquired molecular profiles can be more accurately assigned as a metabolic response of bacteria to the added agent.

The results of minimum inhibitory concentration (MIC) experiments were corresponding to the previous records of our group.^11^ The MIC values of AgLTF complex for SA, EF, and PA were 1.25 mg mL^–1^ (approximately 0.4 mmol L^–1^), 1.25 mg mL^–1^ (approximately 0.4 mmol L^–1^), and 0.625 mg mL^–1^ (approximately 0.2 mmol L^–1^), respectively. It is worth mentioning that the MIC values of Ag^+^ were then 80 μg mL^–1^ (approximately 0.74 mmol L^–1^), 80 μg mL^–1^ (approximately 0.74 mmol L^–1^), and 20 μg mL^–1^ (approximately 0.19 mmol L^–1^) for SA, EF, and PA, respectively.

### 2.2 Assessing Data Patterns

**Figure 2:**
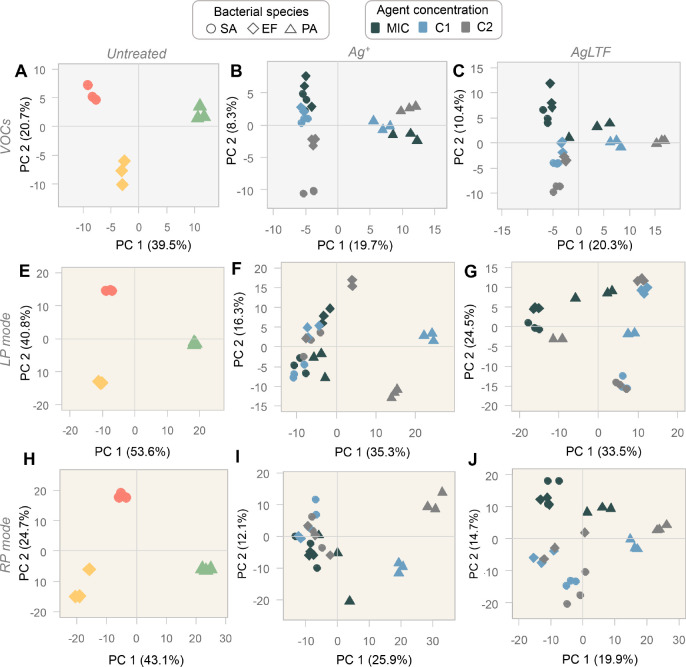
PCA score plots for molecular profiles obtained from GC–MS analysis of VOCs (A–C), as well as MALDI–MS analysis performed using LP (E–G) and RP (H–J) modes. The first, second, and third columns of graphs refer to profile data from untreated, Ag^+^-treated, and AgLTF-treated cultures, respectively. LP = linear positive, RP = reflectron positive, SA = *S. aureus*, PA = *P. aeruginosa*, EF = *E. faecalis*, C1 = 1/2 MIC, C2 = 1/4 MIC.

Principal component analysis (PCA; Figure 2) was used to evaluate patterns within GC–MS and MALDI–MS data, acquired for original and agent-treated cultures. In untreated cultures (Figure 2A,E,H), profiles appeared to be distinctly grouped together according to bacterial species. This confirms the potential usefulness of molecular profiling methods for microbial fingerprinting. The first two PCA components (PC1 and PC2) described approximately 60 and 68% of the total variance for the RP mode and VOCs data, respectively. The distinction between bacterial strains is particularly clear for the LP mode spectra, where PC1 and PC2 were able to explain around 94% of the observed variance. Such outcome is expected, since the referred method is currently well recognized as an accurate proteome-based tool for microorganism identification.^19^ After treatment with Ag^+^, a visible separated segregation of VOC profiles in Gram-positive (SA and EF) and Gram-negative (PA) species appear to occur (Figure 2B). A similar observation can be drawn, but restricted to noninhibitory levels, in case of LP and RP mode profiles (Figure 2F,I). This indicates that milder concentrations of Ag^+^ induce reconfigurations of the metabolome mainly depending on the type of bacterial cell envelope. Gram-positive bacteria are often less susceptible to antimicrobial therapy due to the thickness and composition of their cell wall. In addition, Gram-negative bacteria, due to the negative charge of lipopolysaccharides (LPS) on the cell membrane, suffer with pronounced adhesion and deposition of Ag^+^ onto the cell surface.^20,21^ For AgLTF (Figure 2C,G,J), such divergence between PA assays (at any tested concentration) from other samples also appears to occur for VOC and RP mode data. Additionally, VOCs and RP mode profiles obtained at MIC levels appear to be more distinct from those recorded at lower concentrations. In such cases, PC1 tends to segregate MIC samples from remaining ones—indicating that this is a pattern pertinent mainly to the class of metabolites rather than proteins. The clustering of MIC samples suggests that inhibitory levels cause bacteria to manifest similar metabolomic configurations. It is possible that inhibitory concentrations of AgLTF convert the individual bacterial metabolism to a common primary set of metabolic functions.

Our previous study revealed that pure LTF did not display cytotoxicity in the range from 0.08 to 5 mg mL^–1^. Only the highest tested concentration, that is, 10 mg mL^–1^, caused a decrease in cell viability to about 40%. Here, the measured MICs do not exceed 1.25 mg mL^–1^ —the values almost five times lower than that for pure LTF. The MTT technique—a colorimetric assay—showed for a lowest tested concentration of 0.08 mg mL^–1^, that the complex caused a decrease in cell viability of about 40%, whereas for the corresponding silver nitrate’s Ag^+^ the concentration was two times higher, as well as for pure LTF. It suggested the synergy between Ag^+^ and LTF in complex, and that LTF may promote Ag^+^ entry into cells. The release of Ag^+^ from complex is partially originated from the weakly bonded ionic form of silver and from AgNPs, created at initial rapid sorption step, which is strongly bonded to glutamic and aspartic acid on the surface of LTF clusters.^8,11^

### 2.3 Investigating VOC Profiles

**Figure 3:**
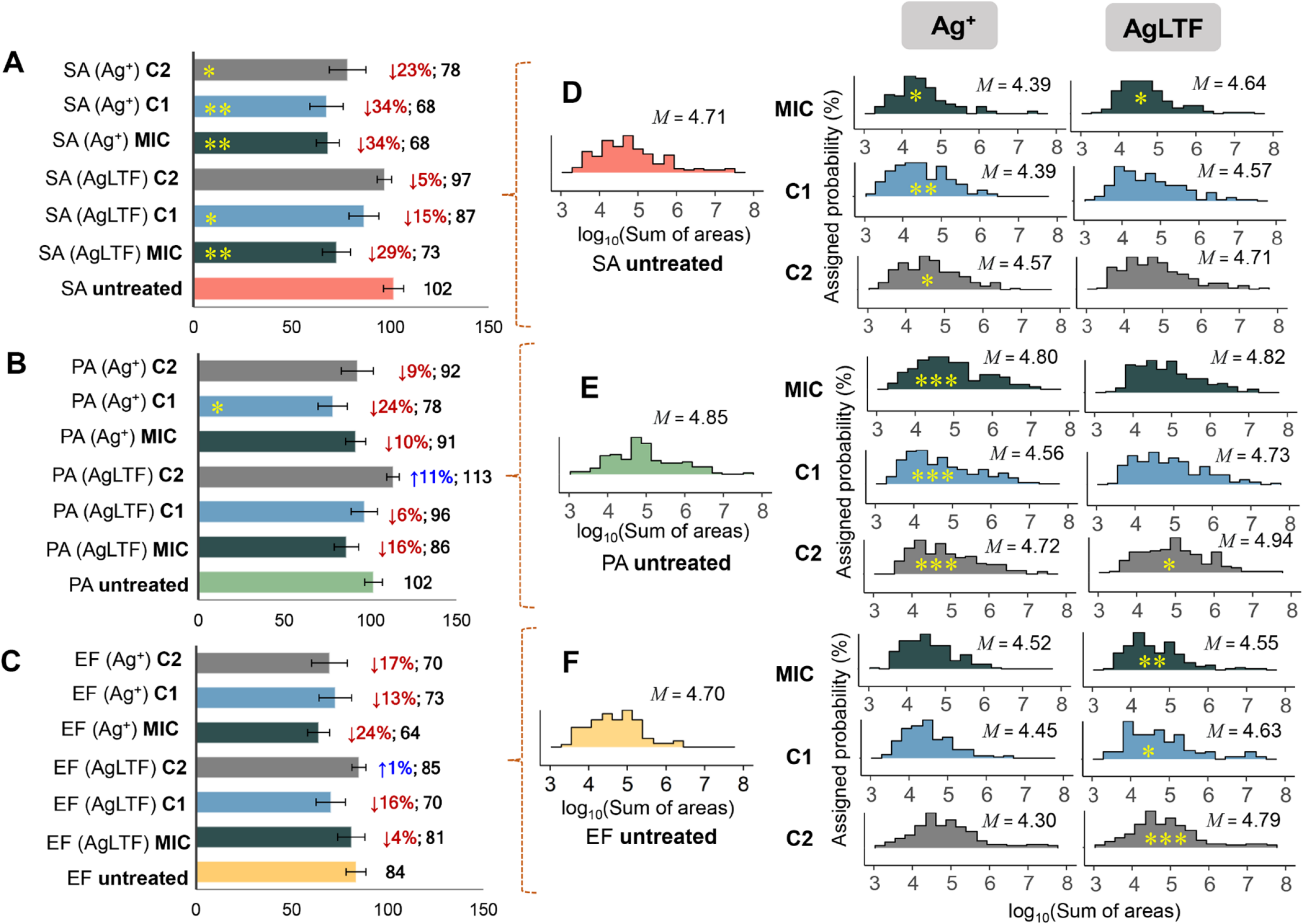
Bar graphs (left part of the panel) present the number VOCs detected in each assay, for (A) SA, (B) PA, and (C) EF. In the right part of the panel are showed density plots for VOC profiles of untreated and treated cultures of (D) SA, (E) PA, and (F) EF. Significance of the change in the number of detected VOCs (A–C) or the total VOC response (D–F), in relation to the corresponding untreated bacteria: *p < 0.05, **p < 0.005, ***p < 0.0005. Error bars refer to the standard deviation observed for metabolite counts between replicates. M = median, SA = *S. aureus*, PA = *P. aeruginosa*, EF = *E. faecalis*, C1 = 1/2 MIC, C2 = 1/4 MIC.

Regarding the VOC profiling analysis, an average total of 102 compounds were detected in unstressed cultures of SA and PA, while 84 different volatiles were found in untreated cultures of EF (Figure 3A–C).

For all species, Ag^+^ provoked a decrease in the number of generated volatiles, regardless of the added concentration of the agent. A greater reduction in the number of VOCs found in the original profile was evidenced in the case of SA (from 23% to 34%), followed by EF (from 13% to 24%). For these species, the most accentuated inhibition of VOCs formation occurred for cultures supplemented at the MIC level. In PA, the decrease in the native set of volatiles ranged from 9 to 24%; however, the greatest inhibition was observed at the C1 level. For the same bacterial species, the overall depletion of VOC profiles caused by AgLTF was slightly lower than in the case of Ag^+^: it ranged from 5% to 29% for SA, 6% to 16% for PA, and 4% to 16% for EF. AgLTF at the concentration level of C2 caused a decrease of 5% in the original VOC counts for SA. On the other hand, for PA and EF, such a concentration slightly augmented the observed variety of volatiles. In such a context, a reduction in the qualitative volatilome of bacteria can be associated with the inhibition of biochemical processes involved in the biosynthesis of certain compounds. As demonstrated, an increase in the concentrations of agents does not necessarily imply the continuous diminishment of bacterial volatilome. Compounds incident at progressively higher concentration levels can be generated through metabolic mechanisms of resistance, which are upregulated at specific degrees/types of stress. In addition, such compounds can be products of catabolic reactions or result of the interaction of different biomolecules with reactive oxygen species (ROS)—overproduced due to the cellular chemical imbalance promoted by the tested agents.^22^ This observation is particularly pertinent in the present case, since C1 and C2 are below the inhibitory threshold. Therefore, such concentrations are likely to stimulate metabolic shifts aiming stress compensation, which assures bacterial growth and development.

**Figure 4:**
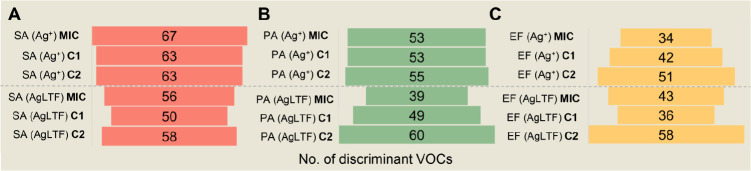
Bar graphs showing the number of discriminant VOCs (*p* < 0.05) in (A) SA, (B) PA, and (C) EF, when comparing treated cultures with corresponding untreated bacteria. SA = *S. aureus*, PA = *P. aeruginosa*, EF = *E. faecalis*, C1 = 1/2 MIC, C2 = 1/4 MIC.

Density plots (Figure 3D–F) represent the distribution of average VOC response, for each of the performed assays. The addition of Ag^+^ in the medium led to a reduction in the total relative response of VOCs in relation to unstressed populations, for all cases. AgLTF provoked a reduction in VOC response only for cultures treated at C1 and MIC, while C2 caused the dislocation of VOC profiles to regions of greater populations (for PA and EF) or kept total VOC response practically unaltered (in case of SA). Hence, for AgLTF, the concentration of VOCs in the HS of cultures tends to increase at C2, thus, suggesting that bacteria treated at lower concentrations of AgLTF increased their metabolic activity, leading to the increased generation of microbial VOCs. In summary, both agents, at higher concentrations, reduced the response of emitted VOCs. VOC metabolites can play essential biological roles in the adaptation of microorganisms. As potential biologically active molecules, such compounds may obey concentration-dependent functions.^23,24^ For this reason, significant alterations in the concentrations of produced microbial volatiles can also represent an active response of the biological system to the altered medium. This aspect was further approached in Figure 4, where it presented the number of volatiles that showed statistically relevant alterations in their responses related to native cultures.

It can be observed that the variation in the number of discriminating features was not directly proportional to the amount of agent added to the medium. Regarding the effects of Ag^+^, SA was the bacteria that suffered the greatest number of significant alterations in relation to control samples. Significant changes induced by AgLTF were more incident at the C2 concentration level, for all bacteria. According to what was demonstrated in Figure 3, most of such profile alterations refer to positive modulations of VOC metabolites. This last approach also suggests that EF was the bacteria less influenced by the studied agents, once they displayed the lower number of significantly affected metabolites.

**Figure 5:**
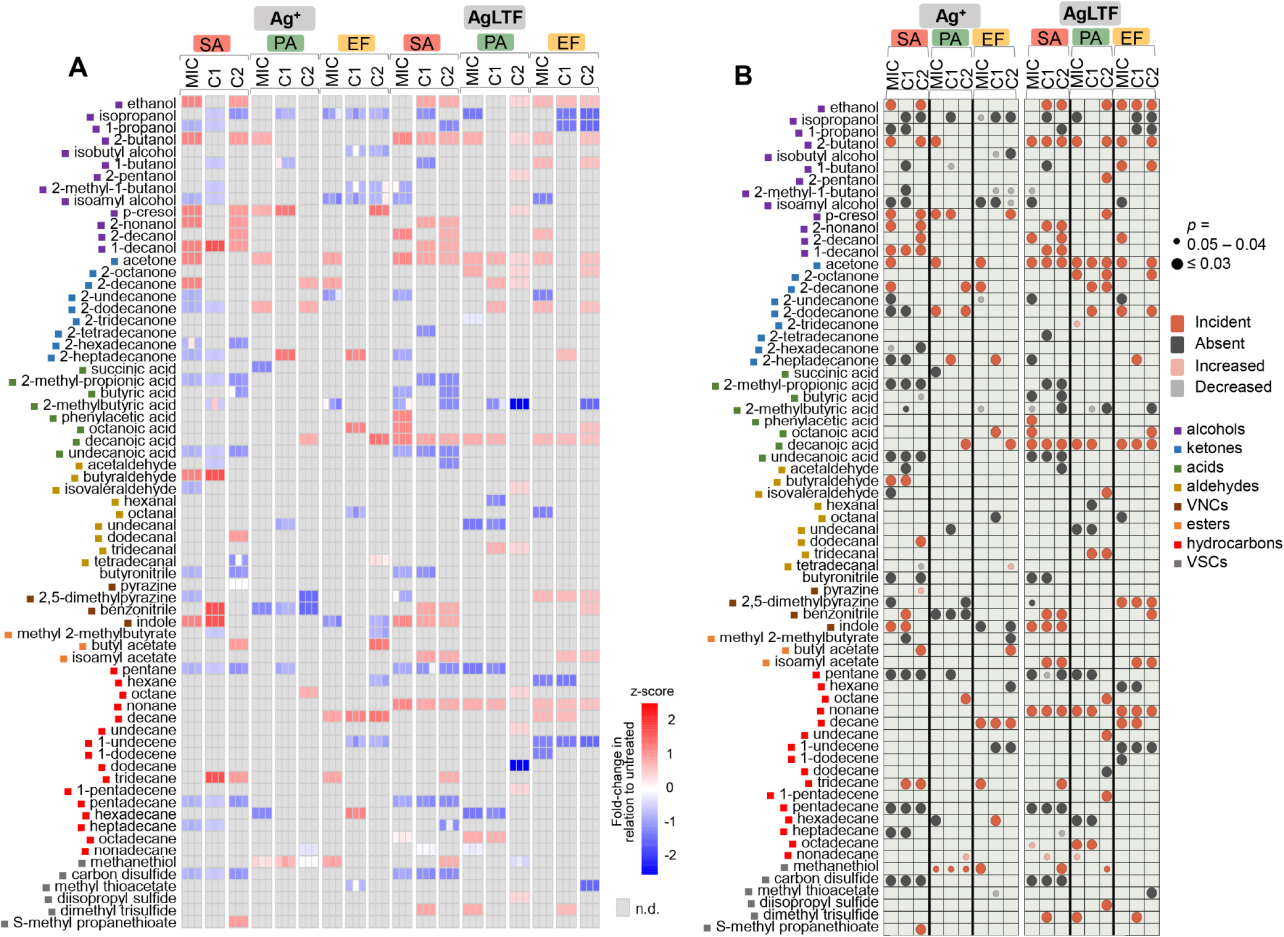
(A) Heatmap showing the FC in the response of main discriminant VOCs in relation to the corresponding untreated bacteria, for each of the assay replicates with Ag^+^ (left part) and AgLTF (right part). (B) Chart summarizing the trend presented by main discriminant VOCs in face of Ag^+^ (left column) and AgLTF (right column) supplementation. The color of the dots and their sizes, respectively, refer to the nature of the observed change in relation to untreated samples and its significance (*p* value). VNCs = volatile nitrogen compounds, VSCs = volatile sulfur compounds, n.d. = not detected, SA = *S. aureus*, PA = *P. aeruginosa*, EF = *E. faecalis*, C1 = 1/2 MIC, C2 = 1/4 MIC.

A detailed analysis of the changes in VOC responses is shown in Figure 5, which shows VOC fold-changes (FC) for all replicates (Figure 5A) and a summary of the results of statistical comparisons with untreated bacteria (Figure 5B). In the mentioned chart, only discriminant compounds (considering untreated cultures against those treated at different concentrations of agent), which can be ascribed as possible bacterial metabolites are displayed. Additionally, these VOCs appear grouped according to their chemical classes. Most of the induced alterations seem to involve alcohols, FA, and hydrocarbons.

For Ag^+^-treated samples, the disappearance or decrease of compounds (prevalence of grayish dots) prevails. On the other hand, metabolic shifts induced by AgLTF were more diverse and included the appearance of several metabolites not previously detected in the unstressed samples (greater prevalence of reddish dots). Regarding the VOCs negatively modulated by Ag^+^, some of these can be ascribed as typical products of the keto acid (KA) pathway. Isovaleraldehyde, absent in Ag^+^-treated SA, is yielded by the decarboxylation of 2-KA derived from leucine. Aldehydes generated by the mentioned decarboxylation of 2-KA may be reduced to primary alcohols, through the action of alcohol dehydrogenase. 1-Butanol and 2-methyl-1-butanol are bacterial fusel alcohols ascribed to this pathway, both of them found negatively modulated by Ag^+^ in SA.^14^ The mentioned alcohols may be converted to esters by the alcohol acetyltransferase enzyme, which catalyzes the transfer of acetyl from acetyl-CoA to the substrate. In this manner, 1-butanol and 2-methyl-1-butanol give rise to butyl acetate and isoamyl acetate, respectively.^14,25^ These esters became incident after supplementation with Ag^+^ (butyl acetate) and AgLTF (isoamyl acetate), for lower concentrations of agents (C1 and C2).

The generation of fatty aldehydes by microbials can occur in connection with fatty acid biosynthesis, being dependent on the action of reductase enzymes on fatty acyl-CoA/ACP units.^26^ The observation of decreased fatty aldehydes (octanal and undecanal—in PA and EF, respectively) may be evidence on the inhibition of the mentioned reductases. In SA, two methyl ketones—2-dodecanone and 2-hexadecanone—became absent due to Ag^+^ treatment, indicating the hampering processes of decarboxylation of β-KAs.^27^ Besides that, the depletion of few hydrocarbons existing in the corresponding untreated cultures (e.g., pentane, hexane, pentadecane, heptadecane, 1-undecene, 1-dodecene), particularly after Ag^+^ supplementation was noticed.

Alkanes with long straight carbon chains are possibly derived from the decarbonylation of fatty aldehydes, promoted by the acetoacetate decarboxylase enzyme.^28^ Terminal alkenes can be generated by the action of fatty acid decarboxylase on FA obtained after the elongation phase.^14^ Considering the exposed, the depletion of aforementioned processes can reflect a general inhibition of enzymes participating in different stages of FA pathway in bacteria.

Conversely, other alkanes were detected only after agent supplementation, namely, tridecane (for both agents, in SA and EF), undecane (for AgLTF, at C2), octane (at lower concentrations, for both agents) in PA, as well as decane in EF (for both agents, at lower concentrations). In addition, octadecane (in SA) and nonadecane (in SA and PA) are long-chain hydrocarbons, which were positively modulated after agent supplementation. Octane can be released during oleic acid peroxidation followed by α or β cleavage;^29^ Available evidence indicates that tridecane, undecane, and decane emitted by bacteria can play a role as signal mediators between colonies and the environment. Such microbial alkanes promoted an induced systemic resistance in *Arabidopsis* against pathogenic bacteria.^30^

Both agents, but especially Ag^+^, caused the positive modulation of methanethiol—an intermediate of sulfides biogenesis.^31,32^ Methanethiol can be formed primarily through methionine degradation and sulfide methylation. AgLTF triggered the production of diisopropyl sulfide and dimethyl trisulfide by the studied pathogens. These sulfides may be generated through the auto-oxidation of methanethiol.^31^ It is believed that sulfides play a role in bacterial stress resistance—the underlying mechanism possibly relies on the removal of ROS from the medium through chemical inactivation.^33^ Methyl thioesters can be derived from the methanethiol reaction with acyl-CoAs coming from short-chain FA and branched-chain amino acids catabolism. Nevertheless, the biological role of thioesters remains unknown.^34^ The EF metabolite, that is, methyl thioacetate, was negatively modulated by both agents. In SA, *S*-methylpropanethioate became available at the lowest Ag^+^ concentration (C2).

In addition, studied antimicrobial agents provoked significant alterations in the response of several metabolites connected with fermentation processes. A remarkable effect of AgLTF on bacteria, observed at lower extension for Ag^+^, was the positive modulation of ethanol, acetone, and 2-butanol. In the first stage, the pyruvate obtained from glucose can be decarboxylated and reduced to acetyl-CoA molecule. Acetyl-CoA is then converted to acetaldehyde by a CoA-dependent acetylating acetaldehyde dehydrogenase. Finally, acetaldehyde is reduced to ethanol by acetaldehyde dehydrogenase. Acetyl-CoA can be also condensed to acetoacetyl-CoA.^32,35^ The latter can be enzymatically converted to acetoacetate, which can be decarboxylated to produce acetone. Alternatively, acetoacetate can undergo a series of five reactions comprising steps of reduction and dehydration, generating 1-butanol (butanoate metabolism).^36,37^ If fermentation occurs in the medium deprived of oxygen, then acetoin can be produced as a metabolite. This product can be subsequently reduced and dehydrated to 2-butanone, which may be again reduced to 2-butanol.^38^ In general, evidence on the upregulation of the described biochemical paths can be explained by the exhaustion of energetic resources in the bacterial cell. It is known that Ag^+^ ions are able to impair bacterial respiratory chain through the inhibition of participating enzymes. In this sense, it is indicated that silver bound to LTF complex displays this common aspect of the action mode of Ag^+^.

On the other hand, 1-propanol, isopropanol (2-propanol), and isobutyl alcohol (isobutanol) displayed negative trends after the treatment with both agents, a behavior particularly accentuated in case of Ag^+^. Propionibacteria and other anaerobic species can yield propionic acid as the major endproduct of pyruvate fermentation, with propanoyl-CoA as the precursor. Propionic acid biosynthesis occurs naturally through Wood–Werkman cycle and acrylate pathway. Depending on the cultivation conditions, 1-propanol may be obtained at smaller or greater proportion, after a two-step reduction of propanoyl-CoA.^36^ Isopropanol is known as a byproduct of acetone reduction during fermentation, principally among lactic acid bacteria and *Clostridium* species. It was demonstrated that alterations in acetone and 2-propanol proportion may be linked to dysregulation of enzymatic activity. More specifically, augmented specificity of alcohol dehydrogenase for acetone leads to isopropanol accumulation.^36,39^ In KA pathway, longer chain 2-KA serve as precursor units for the amino acid production. Specifically, 2-ketoisovalerate can be converted into isobutyl alcohol.^40^ In this sense, the observed decrease in isobutyl alcohol production after agent supplementation may represent a downregulation of amino acid biosynthesis through the mentioned pathway.

Pyrazine levels increased at C2 in SA after Ag^+^, while 2,5-dimethylpirazyne was depleted after agent addition, particularly in SA. Threonine and tryptophan can be the precursors for pyrazine biosynthesis. Pyrazines are mentioned as potential active microbial metabolites that work in interspecies communication and in a quorum sensing regulator associated with biofilm production.^41,42^ For this reason, agent-induced changes in pyrazine responses can reflect disturbances in bacteria collective behaviors. Indole is probably derived from tryptophan metabolism in bacteria.^41^ Indole is reported to be playing a role in the extracellular signaling in microbial environment, drug resistance, and biofilm formation.^43^ The positive modulation of indole in such context can suggest an ongoing bacterial resistance response.

Regarding specific effects of AgLTF on bacterial volatilome, the expressive incidence of decanoic acid and nonane was also verified. Nonane became incident in the cultures of all bacteria, particularly in relation to higher AgLTF concentrations, possibly indicating ongoing oxidative stress. Likewise octane, nonane can be formed through α and β scission of octanoic acid after its interaction with ROS.^29^ Medium chain FA (MCFAs), like decanoic acid (capric acid), are bacterial metabolites studied in frame of their inhibitory properties against competitor microorganisms. MCFAs effects on oral microbial ecology have been studied and are characterized by the suppression of the colonization and emergence of additional bacteria—the involved mechanisms possibly rely on MCFAs nature in contributing to biofilm formation, evolution, and its dynamics. MCFAs may grant to certain species a competitive edge that allows them to emerge within pathogenic biofilms.^44^ In the present context, the emission of decanoic acid provoked by agents suggests the triggering of mechanisms supporting bacterial survival.

### 2.4 Affected Metabolic Pathways

**Figure 6:**
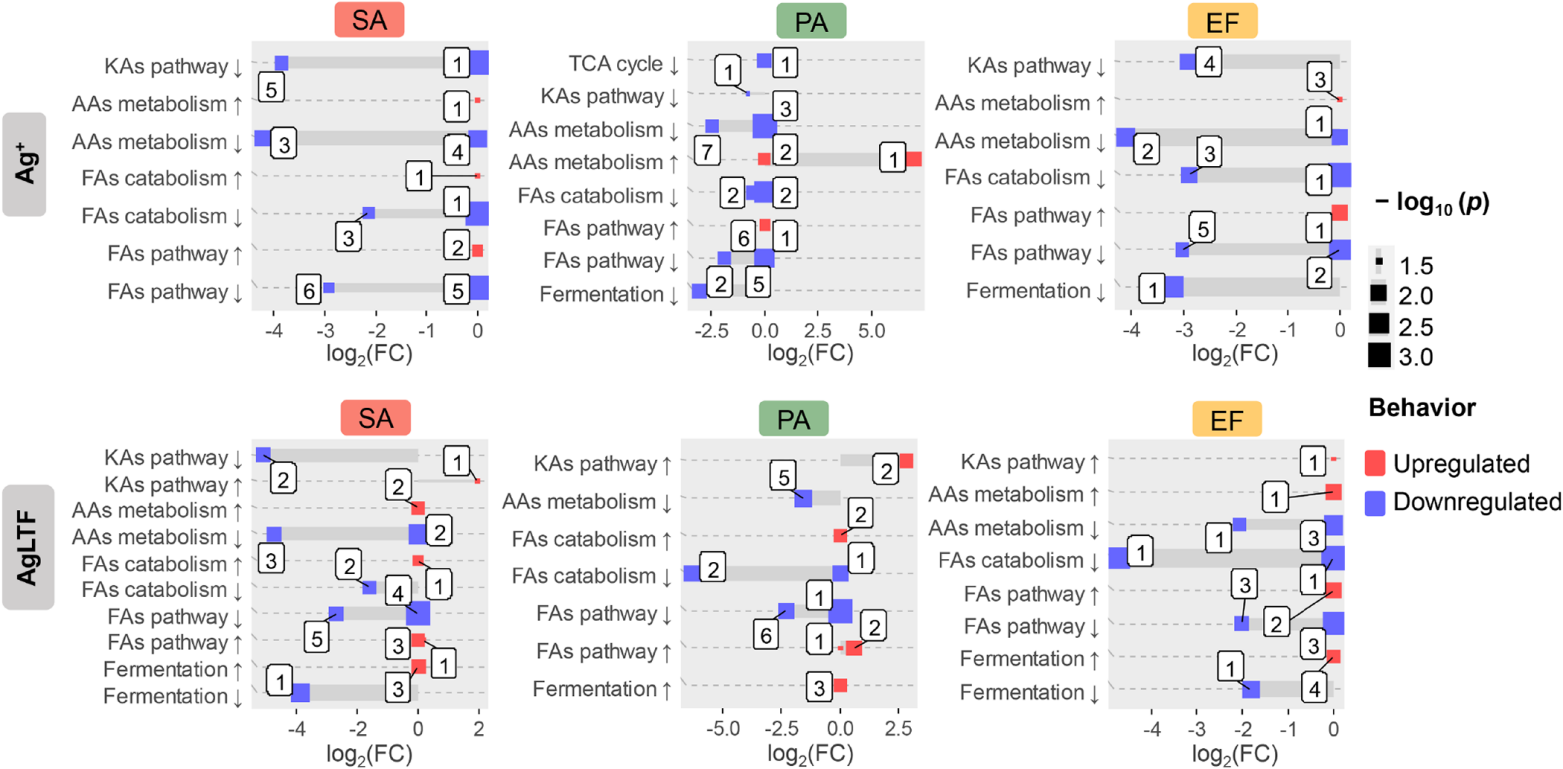
Weighted plots representing significant alterations in VOC metabolites translated as related bacterial pathways, after treatment with Ag^+^ (superior part of the panel) and AgLTF (inferior part of the panel). The numerals inside the boxes indicate the number of VOCs ascribed to a certain mechanism. KAs = keto acids, AAs = amino acids, FAs = fatty acids, FC = fold-change, SA = *S. aureus*, PA = *P. aeruginosa*, EF = *E. faecalis*, C1 = 1/2 MIC, C2 = 1/4 MIC.

Figure 6 depicts the trends of (upregulation and downregulation) exhibited by general metabolic pathways in bacteria, based on volatilome assessment.

This approach encompassed the statistical comparison between untreated cultures versus all cultures treated with different concentrations of agents, for Ag^+^ and AgLTF data sets. In this way, such representation provides the visualization of general remarks on displayed metabolic behaviors induced by the agents, regardless of their concentrations. Ag^+^ caused a more severe inhibition of KA pathway, while AgLTF showed to promote the production of a few KA products. When compared to the AgLTF effect in the same bacteria, Ag^+^ also implied in a more extensive inhibition of FA anabolic pathway, as the number of involved metabolites negatively modulated were much superior to those increase/produced in the medium. While Ag^+^ appears to suppress fermentation pathways, AgLTF enhanced the formation of fermentation products for all bacteria. The activation of fermentative process can signal the depletion of oxygen or impairment of the electron transport chain in bacteria. Regarding FA catabolic pathway, Ag^+^ led to the depletion of a greater number of related metabolites, indicating a reduction in beta-oxidation processes. Unlike AgLTF, Ag^+^ provoked greater modulation of amino acids (AAs) metabolism (e.g., upregulation of the metabolism of sulfur-containing AAs). Silver ions also seemed to be able to influence the second stage of cellular respiration in Gram-negative bacteria, as evidenced by the decline in succinic acid in PA—a tricarboxylic acid (TCA) cycle intermediate.

**Figure 7:**
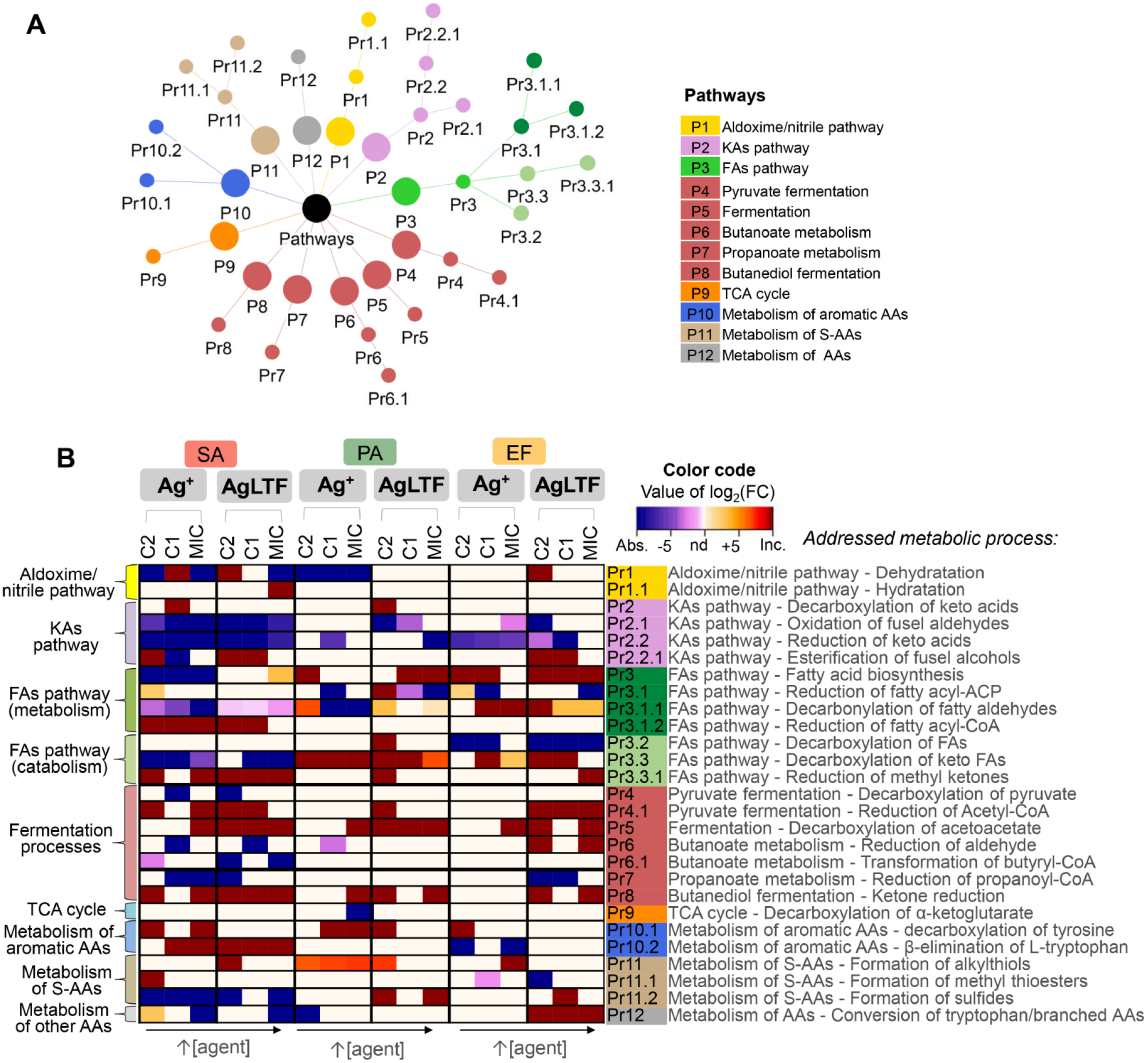
(A) Network displaying connections between possibly affected pathways. (B) Heatmap depicting the main variations in ascribed specific metabolic processes in relation to unstressed cultures. The enumeration of processes (Pr) refers to their position in the metabolic path, that is, dependency with the previous step. FC = fold-change, Abs. = absent (pertaining VOCs were detected only in untreated samples), nd = not detected, Inc. = incident (pertaining VOCs were detected only in treated samples), KAs = keto acids, FAs = fatty acids, ACP = acyl carrier protein, CoA = coenzyme A, TCA = tricarboxylic acid, AAs = amino acids, S-AAs = sulfur-containing amino acids, SA = *S. aureus*, PA = *P. aeruginosa*, EF = *E. faecalis*, C1 = 1/2 MIC, C2 = 1/4 MIC.

Next, the VOCs which presented a concentration-dependent significant change were ascribed to specific metabolic reactions that would be involved in their formation. A network showing the interrelations among the candidate altered pathways is showed in Figure 7A, while a heatmap displaying the alterations occurred for each metabolic process is showed in Figure 7B.

The aldoxime/nitrile pathway occurs in plants and microorganisms, and is responsible for AAs conversion to nitriles, where aldoxime dehydrogenase is a key enzyme. Nitriles are reported to be acting in biological self-defense mechanisms. The formation of nitriles (Pr1) was decreased by both agents, at the MIC level, for SA and PA, but increased for SA and EF, at lower concentrations. This demonstrates the achievement of an agent threshold that triggers the conversion of potential substrates to produce nitriles as a resistance response. Alternatively, or at the MIC, pathway products are reduced through a possible inhibition of cytochrome P450 (CYP) involved in aldoxime formation. Nitriles can be hydrolyzed to acids and nitrogen compounds (Pr1.1), which are incorporated in carbon and nitrogen metabolism.^45^ This ultimate step was induced in SA at higher AgLTF concentrations. Some nitriles, such as benzonitrile, demonstrate to induce nitrilase enzyme, elevating the levels of hydrolysis products.^46^

KA pathway in microorganisms is a path for AA catabolism and can serve as an alternative route for energy obtaining by means of reduced nicotinamide adenine dinucleotide (NADH) regeneration.^47^ One of the first steps of KA metabolism—the decarboxylation of the AA-derived α-KAs resulting in aldehydes (Pr2)— was enhanced by both agents, at lower concentrations. The next two stages of the pathway (Pr2.1 and Pr2.2) were negatively modulated by both antimicrobials, especially in SA. The oxidation of fusel aldehydes results in carboxylic acids—such mechanism may have protective functions in bacterial cell, once it can promote ROS detoxification. The derived carboxylic acids may also contribute to the cell signaling program for ROS management in bacteria, which could support the observed depletion of such products after agent’s supplementation.^48,49^ The reduction of the same fusel aldehydes is implied in the production of alcohols and NADH, with the first being able to undergo esterification.^25^ Metabolites linked to this last process (Pr2.2.1) were mainly augmented at C2 and/or C1 in Gram-positive bacteria, which can be linked to the decline of the metabolites formed in the previous steps, indicating the induction of ester formation. Metabolic reactions naturally yielding esters appear to promote detoxification of aldehydes in the medium and the recycling of cofactors, therefore, playing physiological roles in bacteria. Some microbial estersare also related to cross-species communication.^50^

Ag^+^ caused inhibition of fatty acid biosynthesis in SA. In other assays, FA levels were augmented. In all cases, AgLTF at the MIC level demonstrated the enhancement of the generation of free FA (FFAs). This indicates even at inhibitory concentrations, that AgLTF was not able to impair MCFA synthesis and may even prompt resistance mechanisms based on such molecules. The decarbonylation of fatty aldehydes, that is, the formation of alkanes, appears to be a metabolic process that is more sensitive to agent concentration. The increase in Ag^+^ and AgLTF levels caused decreased total responses of products of this reaction in both SA and PA, while the same parameter was consistently increased for EF bacteria. Many studies have found that *E. faecalis* exhibits strong resistance to both Ag^+^ and AgNPs.^51^ The reduction of fatty acyl-CoA, which gives rise to a primary alcohol, was a mechanism specifically induced in SA, by both agents. The decarboxylation of keto FAs (Pr3.3), resulting in the production of methyl ketones, was progressively inhibited as agents’ concentration augmented in SA. In the remaining bacteria, this metabolic step was induced at lower concentrations (C2, mostly); then, methyl ketones response was lowered as Ag^+^ and AgLTF levels were elevated (C1/MIC). A ramification of such process, the reduction of produced methyl ketones to form the respective alcohols (Pr3.3.1), was enhanced in SA. Hence, the agents promoted fatty acid catabolism in the sense of the formation of secondary alcohols.

Several biochemical steps involved fermentative pathways were induced by the agents: the reduction of acetyl-CoA (Pr4.1), the decarboxylation of acetoacetate (Pr5—for Ag^+^, solely at MIC level), and butanediol fermentation (Pr8). Contrarily, butanoate and propanoate metabolisms were, in general, reduced (Pr6, Pr6.1, and Pr7). As mentioned, one of the Ag^+^ mechanisms of action comprises the binding to the sulfhydryl group of main respiratory chain proteins, causing disabled cellular respiration.^14^ Therefore, aerobic fermentation was principally intensified by agent supplementation, probably because it is more energy efficient. This possibly decreases pyruvate availability, causing obstruction of butanoate and propanoate pathways. Regarding butanediol fermentation (pyruvate–diacetyl–acetoin pathway), it occurs in aerated medium because the dissolved oxygen fraction is limiting, with the lessened oxygen supply rate elevating 2,3-butanediol yield.^52^ Through the activity of diol dehydratase, followed by alcohol dehydrogenase enzyme, butanediol can then be transformed into 2-butanol^53^ —the VOC indicative of butanediol pathway induction in the present study.

Succinate became undetected after Ag^+^ addition at the MIC level, in PA (Pr9). Studies on *Escherichia coli* show that this metabolite can be obtained by the reduction and oxidation branches of the TCA cycle and the glyoxylate pathway, which generate succinate directly from isocitrate. The oxidative and reductive routes of TCA cycle tend to remain active under anaerobic and aerobic conditions, respectively.^54^ Succinate dehydrogenase is associated with the conversion of succinate to fumarate, which is induced aerobically. The succinate–fumarate couple may serve both as an oxidant and reductant for the cell respiratory chain.^55^ Therefore, succinate depletion in the medium can be related to enhanced electron transport from metabolites to fumarate, which is coupled to ATP synthesis once it can be used as an oxidant by oxidative phosphorylation. The aromatic AAs, l-phenylalanine, l-tyrosine, and l-tryptophan, belong to the family of α-AA ubiquitously involved in the synthesis of proteins. Tyrosine decarboxylase genes have been encoded in the genome of several bacterial species in the genera *Lactobacillus* and *Enterococcus*. This enzyme is able to decarboxylate l-tyrosine into tyramine, which plays a role in maintaining the pH homeostasis in bacteria, such as *E. faecalis*.^56,57^ In our study, the decarboxylation of the tyrosine pathway was inhibited in the case of SA and EF when compared AgNPs versus AgLTF. For PA, inhibition of the pathway was noticed for the concentration of MIC and C1 (Pr10.1). Hydrolytic β-elimination reaction catalyzed by tryptophanases leads to the formation of indole from L-tryptophan.^58^ For SA and EF, the process β-elimination were enhanced after the addition of AgLTF. Remarkably, no effect of silver influence was observed for PA (Pr10.2). The metabolism of sulfur-containing AAs was also affected by silver, particularly in the pathway related to the formation of sulfides. In the case of PA and EF, we observed opposite behavior toward the action of silver. For PA, the formation of sulfides was accelerated for AgLTF as compared to no influence of AgNPs in this metabolic process. On the contrary, EF manifested the opposite relationship (Pr11.2). The biogenesis of H_2_S has been mainly attributed to the transsulfuration pathway. This involves the activity of two enzymes in the pathways named cystathionine beta synthase and cystathionine gamma lyase, in which the production of H_2_S is the result of the transsulfuration pathway (conversion of homocysteine to cystathionine) and 3-mercaptopyruvate sulfurtransferase/cysteine aminotransferase pathway.^59^

The advantage of using AgLTF complex against pathogenic bacteria is the combined antimicrobial action of Ag^+^, AgNPs, and LTF. The beneficial contribution of LTF itself consists mainly in inducing an iron-deficient environment that limits growth of bacteria.^60^ LTF counters different important mechanisms evolved by bacteria to infect and invade the host. For example, in enteropathogenic *E. coli* (EPEC), it was demonstrated that holo-hLf inhibits bacterial adhesion to HeLa cells.^61^ Also, this glycoprotein is able to degrade virulence proteins (such as IpaB and IpaC secreted by *Shigella*), released by infected human cells as protective action against bacterial invasion. Similar effects were observed for *Escherichia coli*, when LTF initiated loss and degradation of several type III secretion proteins (EspA, EspB, and EspC).^62^ Also in the case of *Haemophilus influenzae*, LTF was cleaving two proteins of Gram-negative bacteria, IgA1 protease and Hap, known as autotransporters.^63^ Another example of interfering of LTF on bacterial proteins is enhanced damage of the permeability of the bacterial membrane of Gram-negative bacteria caused by the interaction of the protein fraction of LTF (its structure has cationic areas) with the A lipid of the lipopolysaccharide (anionic character) and its subsequent neutralization.^62,64^ Thus, LTF can hinder bacterial virulence mechanism by interference with adhesion by binding with LPS. Other LTF properties, not strictly related to protein fraction, involve the inhibition of biofilm formation through iron sequestration and promotion of the release of proinflammatory mediators in host cells, including cytokines (IL-1, IL-6, IL-8, IL-12 and TNF-α), lipid mediators, and ROS.^64^ However, the effects of LTF on metabolic pathways remain to be further investigated.

Silver ions used in this study as secondary agents versus AgLTF complex have similar mode of action to AgNPs but stronger antibacterial activity than AgNPs. Silver ions interact with the bacterial cell envelope and destabilize the membrane, modify the inside bacterial cell structure (e.g. nucleic acids and enzymes), and finally, initiate the production of ROS.^65^ On the other hand, AgNPs, as vital components of AgLTF complex, act against bacteria in the following manner: (i) damage of the membrane and alteration of transport activity, (ii) inhibition of cell wall formation, (iii) modulation of the cellular signal system and induction of oxidative stress, (iv) prevention of replication of DNA, cell division and respiratory chain processes, and (v) inhibition of cell growth caused by dephosphorylation of protein substrates.^16^ Our previous studies have presented metabolic changes in bacteria upon treatment with silver ions and AgNPs with a focus on alternations in volatile profiles and protein signatures and depicted bacterial pathways supposed to be involved in the formation of metabolites.^14,16^

### 2.5 Protein and Metabolic Profiles Assessed using MALDI–TOF MS

**Figure 8:**
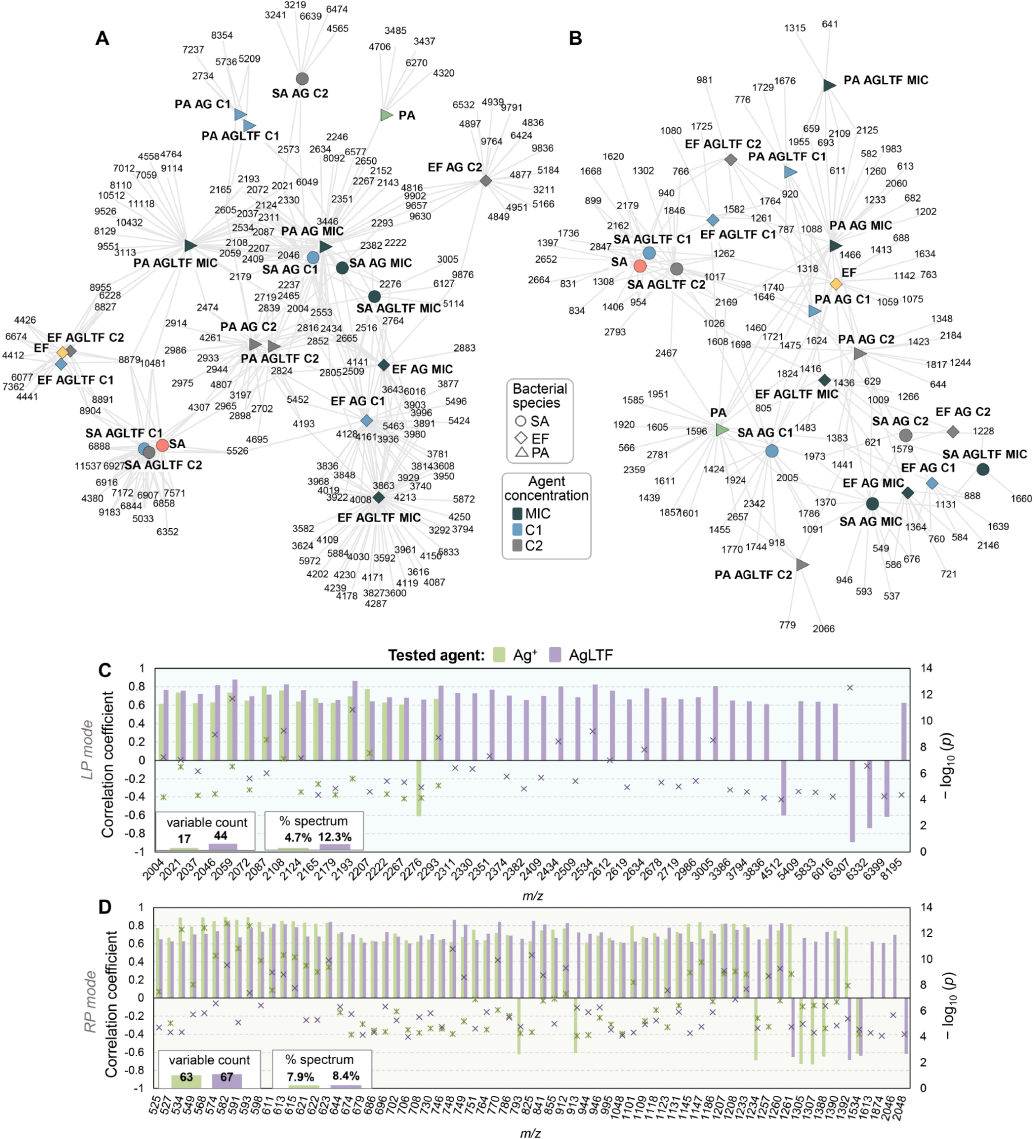
Network showing the most intense discriminant ions found in each assay, considering spectra obtained in the (A) LP and (B) RP modes. These are ions which average intensities were 4 times (in the case of LP) or 25 times (in the case of RP) higher in a given assay in comparison to all others. Bar graphs representing the correlation coefficients calculated when relating ion intensity and agent concentration, for LP (C) and RP (D) mode data. Only ions displaying a strong correlation (rho ≥ |6|, *p* < 0.05) are displayed.

Within the profiles of proteins and metabolites obtained using LP and RP MALDI–TOF MS modes, it could be observed several ions exhibiting differentiated intensities when comparing a given assay with the remaining ones. The relation between the ions which varied the most across the assays is presented in Figure 8A (for LP mode) and Figure 8B (for RP mode). This approach allows to identify the main ions characterizing the spectra of bacteria in the untreated form and after treated with different concentrations of agents.

Pearson correlation analysis was performed aiming to relate ions from MALDI–TOF MS spectra and the different concentration levels of agents that were tested. Assuming as criteria Spearman’s rho ≥ |6| and *p* < 0.05 to consider a variable (ion) as a significant concentration-dependent indicator, 4.7% and 12.3% of the LP mode spectrum appeared to respond to agent dosage for Ag^+^ and AgLTF, respectively. In case of RP mode spectra, a greater number of ions were found to be strongly correlated with both Ag^+^ and AgLTF, showing that this type of profile is more responsive to the antimicrobial dose (Figure 8C,D).

Therefore, AgLTF possibly interferes on protein fraction in a concentration-dependent manner at a greater extension than Ag^+^. In both situations, the strongest significant correlations were positive, indicating that the supplementation with agents led to an increase in the expression of such proteins and lower molecular weight compounds. Indeed, ribosomal proteins might have a role in the development of bacterial resistance. Particularly, specialized ribosome protection proteins hinder a permanent change to the ribosome in order to rescue the translation apparatus from antibiotics inhibition. Their ribosomal protective function and significance are extensively investigated to develop novel translation inhibitors.^66^

**Figure 9:**
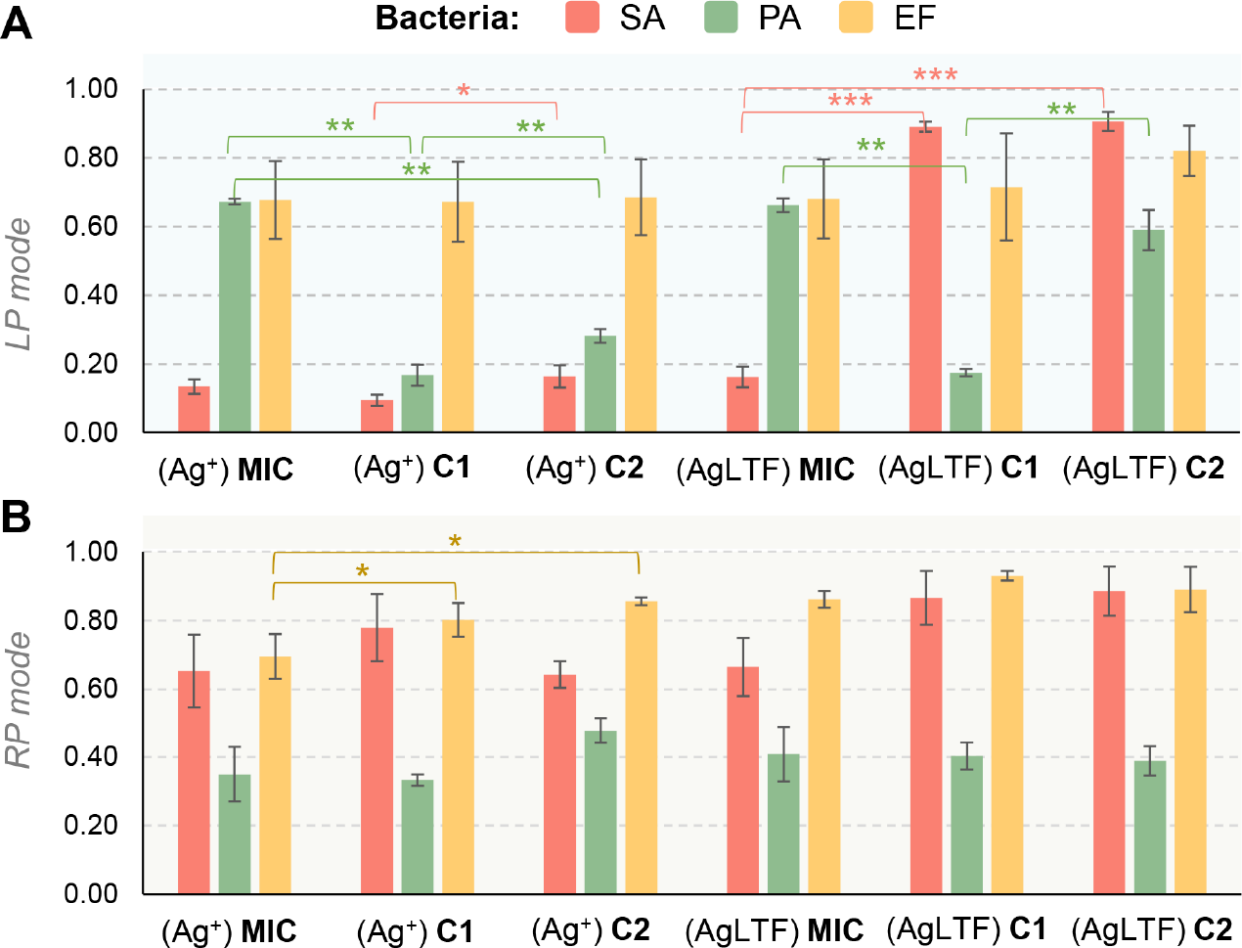
Comparison between average spectrum similarity scores (SSSs) obtained for (A) LP and (B) RP mode spectra, calculated in relation to unstressed samples. Only significant differences are depicted. *0.05 < *p* < 0.001, **p < 1 × 10^–5^, ***p < 1 × 10^–7^ (*T-* test), LP = linear positive, RP = reflectron positive, SA = *S. aureus*, PA = *P. aeruginosa*, EF = *E. faecalis*, C1 = 1/2 MIC, C2 = 1/4 MIC.

Finally, the spectrum similarity score was calculated considering as reference spectrum the MALDI–MS profiles obtained from untreated cultures (Figure 9). For the LP mode, EF protein profile was the most conserved, showing minimum changes in the patterns between modified and untreated bacteria, regardless of the used agent. SA spectra suffered the greatest changes, especially by Ag^+^, but also by AgLTF at the MIC concentration. For concentrations two and four times lower (C1 and C2), the changes caused by AgLTF were minimal. These observations remain consistent with the results obtained from GC–MS experiments. As portrayed in previous graphs, volatile profiles of SA were the most affected among others and demonstrated the greatest reduction of the number of VOCs by both agents (Figure 3), the highest number of discriminating features (Figure 4), and the largest number of variations in ascribed specific metabolic processes in relation to unstressed cultures (Figure 7). The influence of Ag^+^ and AgNPs against SA, notably methicillin-resistant SA and vancomycin-resistant strains, has been excessively investigated.^67^ As mentioned, EF exhibits strong resistance to both agents,^51^ and SA is the bacterium more susceptible to their bactericidal action than PA,^68^ with only short-term protective response to exposure to Ag^+^.^69^ Moreover, it was revealed a greater activity of nanoparticles against SA with a smaller diameter, particularly ranging from 0 to 20 nm. When the nanoparticles were larger than 20 nm, the MIC values were approximately 2.5 times larger than those for smaller ones.^67^ In our case, for used AgLTF complex, the presence of mainly smaller nanoparticles with sizes <20 nm was confirmed.^11^ The overall resistance of volatile and protein profiles of EF also remains in consistent dependence.

On the other hand, RP mode, operating within the range of 500–4000 *m*/*z*, was confirmed to be suitable for bacteria fingerprinting as a complementary approach with manifested correlation between the latter and linear positive mode.^18^ As stated, the main advantage of using the RP mode is the analysis of specialized metabolites, such as low-molecular-weight compounds, from a limited mass range and mutual complementation of information with the linear mode. Here, forthe RP mode, a greater spectrum conservation was observed in comparison to the LP mode. For this type of spectra, the behavior of Ag^+^ or AgLTF appeared more similar. Interestingly, PA protein profiles showed the highest susceptibility to both agents, regardless of applied concentration. SSSs for PA oscillated around 0.4, while for the remaining bacteria was for all cases more than 0.6. PA is the only Gram-negative bacteria in the tested set. The increased susceptibility to silver by Gram-negative bacteria is often addressed to action of AgNPs that are a component part of AgLTF complex. It was numerously proved that Gram-negative bacteria are more susceptible to the toxic effects of AgNPs because of their lack of the thick peptidoglycan layer present in Gram-positive bacteria, which acts as a protective barrier reducing the penetration of nanoparticles. Moreover, Gram-negative bacteria contain LPS in the cell membrane, which their negative charge promotes adhesion of AgNPs. It is commonly acknowledged that AgNPs smaller than 10 nm can directly alter cell permeability, enter bacterial cells, and cause cell damage.^20,70^ In our study, we observed also for PA a distinct susceptibility to Ag^+^ from AgNO_3_. Once again Gram-negative bacteria are known to be more sensitive to silver ions, mostly due to different mechanisms of silver uptake into the cell. Silver ions enter Gram-negative cells via major outer membrane proteins, especially OmpF (and its homolog OmpC), that facilitates the transport of small molecules (e.g. drugs) across the bacterial outer membrane.^65^ It was showed that a minimal bactericidal concentration (MBC) of Ag^+^ for Gram-positive bacteria was more than 32 times higher than the MBC values for the Gram-negative bacterial cells.^71^

## 3 Conclusions

Although many metabolic effects of the studied agents on bacteria were similar, our findings also highlight AgLTF and Ag^+^ distinct antimicrobial behaviors, which affected metabolic pathways of the chosen bacteria differently. Both agents induced the reduction of acetyl-CoA, decarboxylation of acetoacetate, and butanediol fermentation, but hindered butanoate and propanoate metabolism. The AgLTF complex enhanced the production of diisopropyl sulfide, dimethyl trisulfide (playing the role in bacterial stress resistance), ethanol, acetone, 2-butanol (possibly the exhaustion of bacterial energetic resources) and, expressively, decanoic acid and nonane (as result of oxidative stress). AgLTF also accelerated KAs and fermentation pathways, while Ag^+^ inhibited them as well as the FA anabolic pathway. Our results demonstrated that spectra obtained for the RP mode suffered less changes induced by agents than in the case of the LP mode, showing that bacteria growth inhibition promoted by both agents includes broad effects on the small proteins fraction. It was also noticed that AgLTF possibly interferes on the proteins in a concentration-dependent fashion. The RP mode appeared to be suitable for observing modifications in bacterial metabolism, with a greater number of ions strongly correlated with both agents’ concentration. Both VOCs and MALDI–MS data suggested a higher metabolic susceptibility of SA and PA, while EF molecular profiles were less susceptible. The presented outcomes can provide a deeper understanding of the metabolic responses of bacteria in the case of AgLTF versus Ag^+^, as well as the related potential mechanisms of resistance. The study of the involved molecular mechanisms might be a key aspect in designing more effective therapeutic strategies for managing infections and for further research regarding alternative antimicrobial compounds.

## 4 Materials and Methods

### 4.1 Instruments

The ultrafleXtreme MALDI-TOF/TOF mass spectrometer (Bruker Daltonics, Bremen, Germany) equipped with a modified Nd:YAG laser (smartbeam IITM) operating at the wavelength of 355 nm and the frequency of 2 kHz was used to acquire spectra from isolated bacteria. MALDI–TOF MS spectra were recorded manually in linear positive mode within the range of 3000–30 000 *m*/*z*, and in reflectron positive mode within the range of 500–4000 *m*/*z*. The applied acceleration voltage was 25 kV. Optical density (OD) measurements were performed with a DEN-1B densitometer (Biosan, Riga, Latvia). Incubating Microplate Shaker (VWR International, Radnor, PA, USA) was used for incubation of HS vials with bacterial content. The GC–MS analyses were carried out using an Agilent 6890A gas chromatograph coupled to an Agilent 5975 Inert XL MSD mass spectrometer (both from Agilent Technologies, Santa Clara, CA, USA) associated with an autosampler MPS2 (Gerstel, Sursee, Switzerland). The system was equipped with a DB-624 UI 60 m × 0.25 mm × 1.40 μm column (Agilent, Palo Alto, CA, USA). Compound identification was processed by searching the obtained mass spectrum in the NIST11 mass spectral library. The criterion for peak detection was a signal-to-noise ratio of at least 3 and peak integration was done manually. Spectrum search encompassed baseline subtraction and averaging over a peak. Forward and reverse match quality of at least 750/1000 was considered as the lower match threshold. Peaks detected in the corresponding blanks were deleted from the total data set, to obtain signals attributed solely to bacterial activity. Extractions of VOCs were performed using 65 μm polydimethylsiloxane (PDMS)/divinylbenzene (DVB) fiber (Supelco, Bellefonte, PA, USA).

### 4.2 Chemicals and Materials

Water LC–MS Chromasolv, ethanol, acetonitrile, trifluoroacetic acid, formic acid, and isopropanol were purchased from Sigma-Aldrich (Steinheim, Germany). Ultrapure water from a Milli-Q water system (Millipore, Bedford, MS, USA) was used throughout the study. All chemicals for MALDI–MS analyses were supplied at the highest commercially available purity from Fluka Feinchemikalien GmbH (part of Sigma-Aldrich). Polished steel targets (Bruker Daltonics) were used for sample analysis. α-Cyano-4-hydroxycinnamic acid (HCCA; Sigma-Aldrich) was employed as a matrix using dried droplet method for sample and matrix deposition. Bruker Bacterial Test Standard and cesium triiodide (CsI_3_)^72^ standard were used for external calibration (Bruker Daltonics) of spectra obtained in the LP and RP modes, respectively. The HCCA matrix (10 mg mL^–1^) was prepared in a standard solvent (50% acetonitrile, 47.5% water, 2.5% trifluoroacetic acid). HS screw top 20 mL clear vials and magnetic polytetrafluoroethylene/silicon screw caps (18 mm thread) for GC–MS experiments were purchased from Agilent (Santa Clara, CA, USA). The AgLTF complex was obtained from the study of Pryshchepa and co-workers^11^ and applied accordingly in our laboratory. Silver nitrate (AgNO_3_) was purchased from Sigma-Aldrich (Poland). The preparation method for the AgLTF complex consisted of suspending LTF (50 mg/10 mL buffer) in 0.09% NaCl solution at pH = 6. After sonification, 0.5 mL of AgNO_3_ was added, then the content was incubated and centrifuged. Finally, 0.5 mL of supernatant was transferred to a Falcon probe and subjected to Millipore filtration with a molecular mass cutoff of 3 kDa and then diluted in a ratio of 1:10 with 1% HNO_3_. The binding sites of silver ions on LTF were six AA residues, namely, glutamic acid, aspartic acid, cysteine, histidine, arginine, and lysine. It was computed that 50% of silver was bound to LTF, whereas the rest corresponds to reduced or crystallized form of silver.^8^

### 4.3 Bacteria Isolation and Identification

The bacteria used for the investigation, namely SA, PA, and EF (permission of Ethics Committee of NCU in Toruń KB 68/2019), were isolated previously from samples of infected diabetic foot wounds from 16 patients from Provincial Polyclinical Hospital in Toruń (Poland) in the study of Złoch et al. 2021.^73^ Identification of bacterial isolates was performed in the mentioned study using a ultrafleXtreme MALDI–TOF mass spectrometer (Bruker Daltonik GmbH, Bremen, Germany) equipped with the smartbeam-II laser–positive mode. The common formic acid/acetonitrile method of protein extraction was used according to the protocol of the producer of the MALDI Biotyper system (Bruker Daltonik GmbH, Bremen, Germany). Identified bacterial strains were deposited in −80 °C using Microbank (Pro-Lab Diagnostics, Canada)–a unique cryovial system incorporating treated beads and a special cryopreservative solution.

### 4.4 Growth Curves

To draw the growth curves of the three bacteria, the OD of samples was measured using DEN-1B densitometer, which provided results in the unit of McFarland (McF). Nine clean glass tubes were prepared, filled with 10 mL of MHB (Sigma-Aldrich, Germany), in each tube. After sterilization in autoclave at 121 °C, the tubes with cooled content were vortexed for 30 s and the OD of the obtained blanks was measured using DEN-1B densitometer. Then, three loopfuls of bacterial cells were suspended in 1 mL of saline solution to prepare inoculum. Bacterial suspension was thoroughly vortexed for 30 s and the test tubes were inoculated under sterile conditions using 100 μL of the obtained inoculum. Immediately after inoculation, OD at *t* = 0 was determined. Subsequent measurements corresponded to 2, 3, 4, 5, 6, 7, 8, 9, 10, 13, 15, 18, 21, 22, 23, 24, 25, 26, and 27 h of incubation at 37 °C (for PA, the time points at 29 and 35 h were also verified). Growth profiles were assessed for selection of cultivation times to be used in further assays. Such cultivation periods were aimed to refer to the stationary phase of these bacteria, because in this stage the ratio of alive/dead is rather constant, thus, the changes observed in the molecular profiles could be ascribed to a metabolic response to the added stressing agent, minimizing the contribution of metabolic alterations due to the growth process.

### 4.5 Minimum Inhibitory Concentration

The procedure for the determination of MIC for SA, PA, and EF was carried out according to Pryshchepa and co-workers.^11^ The experiment was performed with the utilization of AgLTF complex for which Ag^+^ solution used for production had a concentration of 1200 mg L^–1^. A broth microdilution method was applied with the use of 96-well plates and MHB as a culture medium. The detection was performed based on the fluorescence measurements using a microplate reader (Multiskan, ThermoFisher) and *in vitro* resazurin-based Toxicology Assay Kit (Sigma-Aldrich) according to the protocol provided by the kit supplier.

### 4.6 SPME–GC–MS Analysis

Initially, culture medium (MHB), agent solutions, and vials were autoclaved at 121 °C for sterilization. Vial screw caps were sterilized by being evenly exposed to ultraviolet radiation for 1 h. The samples were prepared on the basis of the results of MIC analysis. For the GC analysis, the samples were prepared in HS vials and the inoculum used was a bacterial culture grown in MHB using Microbank. After inoculation, the bacterial content in vials had approximately 1.0 McF. In this step, three concentrations were tested: MIC value and two noninhibitory concentrations (C1 = 1/2 MIC and C2 = 1/4 MIC). For each of the three studied bacteria, the set of seven samples (made in triplicate) was arranged, namely three for Ag^+^ agent (MIC, C1, and C2), three for influence of AgLTF (MIC, C1, and C2), and one for untreated bacteria. Prepared samples were placed into a stirring incubating microplate shaker at 37 °C and taken out immediately after the optimal incubation times were determined to promote appropriate bacterial growth. In the case of GC–MS analysis, several blanks have been used: untreated cells with medium, pure medium, and the medium treated with the used concentrations of Ag^+^ and AgLTF in order to be paired with all respective positive assays. To favor thermodynamic equilibrium for GC–MS measurements, the samples were preincubated at 37 °C for 30 min. VOC extraction was conducted at 37 °C for 10 min, using a 65 μm PDMS/DVB fiber. Then, the loaded SPME fiber was desorbed into GC inlet port for 2 min. The carrier gas (helium 6.0) flow rate was set as 2.2 mL min^–1^ and inlet temperature was set at 250 °C. The oven temperature program was as follows: the initial temperature of 40 °C was maintained for 4 min, then ramped at 100, 220 (held for 7 min), and 250 °C, at respective rates of 10, 15, and 20 °C min^–1^. The oven was maintained at this last temperature for 5 min. Spectra acquisition was performed within the range of *m*/*z* 25–300, using electron ionization (EI) at 70 eV. Both ion source and the transfer line temperatures were set at 220 °C, while quadrupole analyzer was kept at 150 °C.

### 4.7 MALDI–MS Analysis

For MALDI–MS analysis, the bacterial cultures with approximately 1.0 McF (equal to GC–MS experiments), were prepared also in HS vials. Both silver agents were added and vortexed to obtain the final silver concentration of calculated MIC, C1, and C2 (in triplicate). It meant 18 samples for each bacteria, plus additional three as blanks. The vials were placed in a shaker at 37 °C for 9, 15, or 27 h, which are equivalent to the optimal growth times of EF, SA, and PA, respectively. After that, OD measurements were performed. The biological material submitted to the extraction protocol was a precentrifuged (13 000 rpm/RCF = 15 871*g* for 2 min) bacterial pellet obtained from 1.5 mL of a liquid culture of MHB medium. The extraction protocol was used in the following ways: (i) 300 μL of water was transferred into an Eppendorf tube containing the biological material and mixed; (ii) then 900 μL of 100% ethanol was added to the tube and mixed thoroughly; (iii) this step was followed with centrifugation at 13 000 rpm/RCF = 15 871*g* for 2 min and decantation of the supernatant; (iv) centrifugation was continued for further 2 min and residual ethanol was removed from the pellet with a pipet; (v) subsequently, 5 μL of 70% formic acid was added to the pellet and mixed thoroughly by pipetting and by vortexing, (vi) 5 μL of acetonitrile was added to the tube and mixed carefully; (vii) the whole was centrifuged at 13 000 rpm/RCF = 15 871*g* for 2 min, and 1 μL of the supernatant was spotted onto a polished steel target; (viii) the sample was covered with 1 μL of HCCA matrix solution as soon as the sample spot had dried out; (ix) finally, the sample spot was allowed to air-dry before analysis. All the MS spectra were obtained using the ultrafleXtreme MALDI-TOF/TOF mass spectrometer. All mass spectra were acquired and processed with the dedicated software, namely, flexControl and flexAnalysis, respectively (both from Bruker). The experiments with MALDI MS technique were conducted in triplicate during the study. Spectra were acquired by summing up three individual spectra obtained with 500 laser shots each and were plotted using the Origin software (v. 2015, OriginLab Corporation, Northampton, MA, USA) from raw data without any modifications. The sample preparation protocol for microorganism profiling was performed according to the instructions of mass spectrometer manufacturer.

### 4.8 Data Analysis

Data analysis and visualization was mainly conducted in the R environment (R v.4.2.1), using Rstudio console (v. 2022.02.03, PBC, Boston, MA, USA). The primary VOC data set was organized as a peak table (samples as columns and variables peak areas displayed in rows). MALDI–TOF MS raw spectra in.mzXML format were processed employing “MALDIquant” R package. This package displays a series of functions that can be executed as a pipeline, allowing spectra preprocessing, baseline adjustment, peak detection, and alignment.^74^ Default options were used to perform variance stabilization and spectra smoothing; the baseline was inferred using “SNIP” method, and then subtracted from all spectra (number of interactions = 10 mi). Spectra normalization was executed using “TIC” method. Spectra alignment was carried out under the following parameters: tolerance = 0.2, halfWindowSize = 20, SNR = 3, and warpingMethod = “lowess.” Peak detection considered halfWindowSize = 50 and SNR = 3; peak binding used tolerance = 0.3. Peaks with frequencies lower than 4% were removed. In case of spectra obtained using the RP method, the number of iterations for baseline estimation was equal to 6. For peak detection, halfWindowSize = 200 and SNR = 10 were set. Once the described workflow was concluded, a matrix containing *m*/*z* values and their respective intensities was exported as a Microsoft Office Excel file using “xlsx.”

PCA was performed on scaled data (prepared by subtraction of variable mean and dividing it by the standard deviation) using “prcomp” function. Density plots were built using “ggpubr” package–in this case, input data consisted in the sum of variable peak area followed by common logarithm transformation. A heatmap was prepared with the aid of “heatmap.plus” packages. Additionally, the generation of plots relied on the use of “gplots” and “ggplot2” packages. FC was calculated by dividing the observed variable response in the treated sample by the correspondent response in the untreated sample. “Hmisc” package was employed for the calculation of Spearman’s rank correlation coefficients (rho). SSSs were calculated with the use of “OrgMassSpecR” package, applying the following parameters for LP/RP mode spectra: tolerance = 0.01/0.001, baseline threshold for peak detection = 15.

The difference between the number of detected VOCs and VOC total response in untreated and treated cultures was ascertained using *T*-test. Mann–Whitney U test was selected to assess statistically significant differences in individual VOC responses between control and assay groups (untreated and agent-treated cultures, respectively). Such tests were conducted using IBM SPSS Statistics v.24 (IBM Corp., Armonk, NY, USA). Relevant differences between SSSs obtained for each set of assays were assessed using *T*-test, employing “ggstatsplot” R package.

## Data Availability

The raw chromatographic data underlying this study is available free of charge at the Mendeley Data repository at https://doi.org/10.17632/w8k9yfbwp7.1
